# Evidence that increased azole persistence and stress resistance precede the *in vivo* evolution of azole resistance in *Aspergillus fumigatus*

**DOI:** 10.1128/spectrum.04021-25

**Published:** 2026-03-16

**Authors:** Endrews Delbaje, Laís Pontes, Marcela Savoldi, Sarah Sedik, Karl Dichtl, Martin Hoenigl, Cornelia Lass-Flörl, Cristina Silva Pereira, Angélica Zaninelli Schreiber, Antonis Rokas, Ling Lu, Júlio César Jeronimo Barbosa, Taícia Fill, Thaila Fernanda dos Reis, Gustavo H. Goldman

**Affiliations:** 1 Faculdade de Ciências Farmacêuticas de Ribeirão Preto, Universidade de São Paulo 67782 , Ribeirao Preto, Brazil; 2 Division of Infectious Diseases, Department of Internal Medicine, Medical University of Graz 196266, Graz, Austria; 3 Diagnosic and Research Institute for Hygiene, Microbiology and Environmental Medicine, Medical University of Graz 31475, Graz, Austria; 4 Division of Infectious Diseases and Global Public Health, Department of Medicine, University of California San Diego 31475, La Jolla, California, USA; 5 Clinical and Translational Fungal Working Group, University of California San Diego 8784, La Jolla, California, USA; 6 Institute of Hygiene and Medical Microbiology, Medical University of Innsbruck, Innsbruck, Austria; 7 Instituto de Tecnologia Química e Biológica António Xavier, NOVA University Lisbon, Oeiras, Portugal; 8 School of Medical Sciences - University of Campinas 67791, Campinas, State of São Paulo, Brazil; 9 Department of Biological Sciences and Evolutionary Studies Initiative, Vanderbilt University 5718, Nashville, Tennessee, USA; 10 Jiangsu Key Laboratory for Pathogens and Ecosystems, Jiangsu Engineering and Technology Research Centre for Microbiology; College of Life Sciences, Nanjing Normal University 224704, Nanjing, China; 11 Instituto de Química, Universidade Estadual de Campinas (Unicamp), São Paulo, Brazil; 12 National Institute of Science and Technology in Human Pathogenic Fungi, Natal, Brazil; Stony Brook University, Stony Brook, New York, USA

**Keywords:** *Aspergillus fumigatus*, clinical isolates, azole resistance, azole persistence, secondary metabolites

## Abstract

*Aspergillus fumigatus* is the major causative agent of human aspergillosis. Azoles are the first-line therapy, but resistance is increasing. Here, we characterized voriconazole persistence by screening a global collection of 495 clinical and environmental *A. fumigatus* isolates. We demonstrate two persistence phenotypes: non-growth persisters (NGPs) and slow-growth persisters (SGPs). We focused on nine sequential clinical isolates from a single patient treated with voriconazole for 2 years. Genome sequencing and phylogenomic analyses show that these isolates form a single genetically related population exhibiting within-host diversification. While temporal isolation order does not strictly correlate with genetic relatedness, we observe that increased azole persistence and stress resistance are early adaptive steps in the *in vivo* evolution of azole resistance, preceding the emergence of voriconazole resistance. Our findings indicate that increased persistence and stress resistance may create a permissive adaptive landscape within the host, highlighting the complex evolutionary dynamics underlying antifungal treatment failure.

**IMPORTANCE:**

Azole resistance in *Aspergillus fumigatus* is a major clinical concern; however, treatment failures occur without the presence of classic resistance mutations. This study provides evidence that azole persistence—the ability of susceptible cells to survive drug exposure—and stress resistance precede the *in vivo* evolution of azole resistance in *Aspergillus fumigatus*. We identified two persistence types in a global isolate collection and tracked their evolution in a patient over a 2-year period. Our findings reveal that increased voriconazole persistence and stress resistance emerged before the acquisition of the *cyp51A* resistance mutation. This adaptive phase involved significant transcriptional and metabolic reprogramming, including the upregulation of secondary metabolism. We propose that persistence is not a passive survival state, but an active evolutionary stepping stone that fosters a permissive landscape for the development of resistance. Understanding this “persistence-first” pathway is crucial for developing more effective diagnostics and therapeutic strategies to combat antifungal treatment failure.

## INTRODUCTION

Fungi are one of the most predominant groups of living organisms in the biosphere. They are present across all latitudes and occupy diverse ecological niches, playing essential roles in carbon and nitrogen cycling and interacting with other organisms as symbionts, commensals, and pathogens. Fungal pathogenesis has substantial global consequences, including significant agricultural losses and high mortality in humans and animals due to invasive infections (1). Despite the rising burden of fungal diseases, only a limited number of antifungal drugs are currently available for clinical use. Azoles represent the major class of antifungal agents used in human medicine. Their target is sterol 14α-demethylase (encoded by *ERG11*/*CYP51*), a key enzyme in the ergosterol biosynthesis pathway (2).

*Aspergillus fumigatus* is a saprophytic, thermotolerant fungus that can cause a spectrum of fungal diseases collectively known as aspergillosis (3). The most severe form of aspergillosis is invasive pulmonary aspergillosis (IPA), which affects immunosuppressed patients, those with viral infections or in intensive care, and is associated with mortality rates that can be exceedingly high, particularly in cases involving resistant pathogens (3). The most severe form of aspergillosis is invasive pulmonary aspergillosis (IPA), which affects immunosuppressed patients and has a lethality that can reach 90% (3). The stand-alone first-line therapies against *A. fumigatus* are fungicidal azoles. Although azoles are quite efficient drugs, *A. fumigatus* azole resistance is on the rise. Treatment failure can be associated with azole resistance; however, there is also a lack of success in treating azole-sensitive isolates, which could be attributed to either poor drug absorption or prolonged survival in the presence of an antimicrobial (4–7). Such survival for long periods in the presence of drugs has been well-described in bacteria and is known as persistence or tolerance (8, 9).

Bacterial persistence and tolerance are characterized by the ability of these organisms to survive in supra-minimal inhibitory concentrations (MIC) of antibiotics without acquiring any genetic mutation and while maintaining the original MICs of these antibacterial agents (10–13). Distinguished by their penetrance within a population, antibiotic tolerance and persistence are superficially similar phenomena by which growth-restricted bacteria survive treatment with bactericidal antibiotics (10–12). Bacterial persistence is defined as a sub-population that can survive a drug, while tolerance is defined as a population where every single organism can survive a drug (10–14). Notably, in addition to environmental triggers, mutations can also influence persistence levels—either increasing or decreasing them (15). Although persister and non-persister cells within a clonal population are usually isogenic, certain mutations have been shown to enhance persistence or tolerance (15). In yeast-like fungi, persistence is frequently observed to occur in subpopulations described as a “fraction of growth” (16–18). Certain strains of *A. fumigatus* can exhibit azole persistence, while caspofungin tolerance is a more commonly observed trait across the species (14, 19, 20). Two case reports of hematological patients who developed IPA with treatment failure revealed that the patients were infected with multiple strains, several of which were persisters to voriconazole (VOR) and isavuconazole (6).

Here, we significantly extended a preliminary study (18) that demonstrated VOR persistence in some isolates of *A. fumigatus*. By screening a global collection of isolates from multiple continents, comprising 495 clinical and hospital environmental strains, and evaluating their VOR persistence, we demonstrate the existence of two distinct VOR persistence phenotypes: non-growth persistence (NGP) and slow-growth persistence (SGP). We further characterized nine clinical isolates, two of which were VOR-resistant, derived from the same patient who had been treated with VOR for approximately 2 years. Genome sequencing, phylogenomic, and haplotype analyses indicate that these nine isolates likely correspond to a single population (serial isolates SI9-1 to -9, with the last two, SI9-8 and SI9-9, being VOR resistant). Notably, we demonstrate that increased VOR persistence (as well as greater resistance to various stresses and a higher frequency of colonies capable of growing on VOR) in this series of isolates evolved prior to the emergence of VOR resistance. Transcriptional profiling, combined with metabolomics, of the clinical isolates SI9-1 (VOR susceptible) and SI9-8 (VOR resistant) revealed increased production of secondary metabolites (SMs), suggesting that epigenetic regulation may be involved in the evolution of these phenotypes. These results suggest that the increased azole persistence and stress resistance are early adaptive steps in the *in vivo* evolution of azole resistance.

## RESULTS

### *A. fumigatus* VOR persistence is composed of NGPs and SGPs

**Fig 1:**
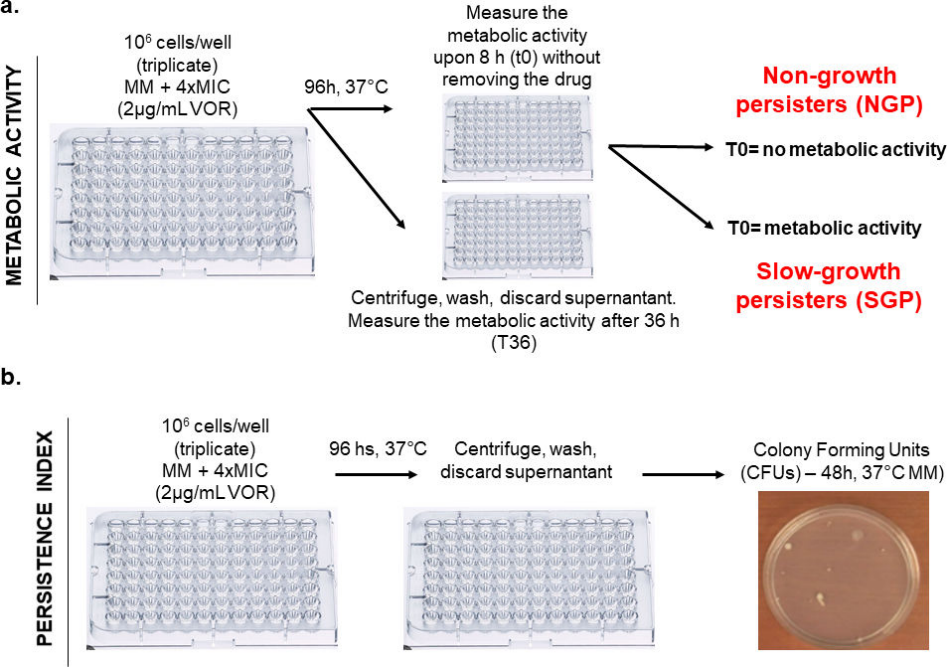
Schematic overview of the experimental design for characterizing NGPs and SGPs. The figure uses representative, non-data images and illustrative icons to visually convey the methodological steps. (**a**) Diagram of the metabolic activity assay protocol. (**b**) Diagram of the persistence index measurement protocol.

**Fig 2:**
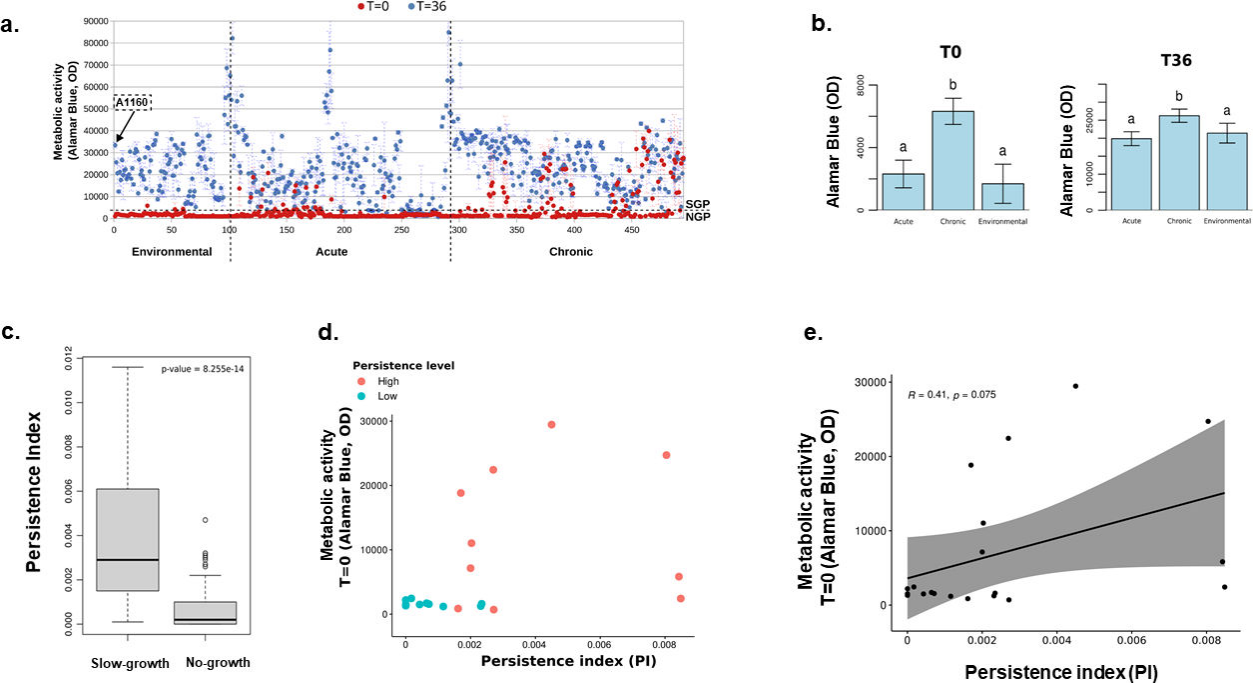
Evaluation of persistence levels in clinical and environmental isolates. (a) Biplot of metabolic activity levels (Alamar Blue) divided into T = 0 and T = 36, showing isolates from environmental, acute, and chronic contexts. The measures of the reference strain A1160 are indicated with a red arrow. The dashed lines represent the threshold of metabolic activity ≤ than 2,000, classifying the SGP and NGP strains. (b) Bar plot with metabolic activity levels comparing isolation contexts, separated by T = 0 and T = 36. Statistical differences determined by post-hoc analyses are shown (*P* < 0.05). Different letters (**a and b**) denote significant differences between groups. The analysis was done using the postHoc package in R. After fitting the linear model, the postHoc function was applied to perform pairwise comparisons. (**c**) Box plots comparing slow-growth and no-growth using the persistence index. Statistical difference determined by Student’s *t*-test (*P* < 0.05). (**d**) Scatter plot of metabolic activity and persistence showing the distribution of the 10 highest and 10 lowest persistence levels. The “high” and “low” refer to the top and bottom 10 strains based on PI, not strictly aligned with SGP/NGP classification. Some high-PI strains may have low T0 values due to recovery after washout. (**e**) Scatter plot of the Pearson correlation level between metabolic activity and persistence of the 10 highest and 10 lowest persistence levels.

We evaluated the occurrence of conidial non-growth or slow growth in the presence of VOR supra-MIC concentrations, measuring metabolic activity using Alamar blue. We arbitrarily defined chronic patient isolates as those isolated from patients with cystic fibrosis and bronchiectasis and acute patient isolates as those from patients with invasive pulmonary aspergillosis, organ transplantation, cancer chemotherapy, and secondary infections; all environmental isolates are derived from patient rooms and the internal hospital environment at Hospital das Clínicas, Unicamp, Brazil (see Table S1 at https://doi.org/10.6084/m9.figshare.31239700). Our sample consisted of 212 chronic patient isolates, 189 acute patient isolates, and 94 environmental isolates, totaling 495 isolates (see Table S1 at https://doi.org/10.6084/m9.figshare.31239700). The VOR MIC for each strain was measured (see Table S1 at https://doi.org/10.6084/m9.figshare.31239700), and 1 × 10^6^ conidia from each strain were incubated at 4-fold the minimal inhibitory concentration of VOR (4× MIC, Table S1 at https://doi.org/10.6084/m9.figshare.31239700) for each strain for 96 h at 37°C. After this period, Alamar blue was added in the presence of VOR, and the strains were allowed to grow for an additional 8 h (T0), or VOR was washed out, minimal medium (MM) was added, and the strains were allowed to grow for an additional 36 h (T36; Fig. 1a; see Table S1 at https://doi.org/10.6084/m9.figshare.31239700). At T0, metabolic activity was measured after 96 h of incubation with 4× MIC voriconazole to distinguish between strains that can grow slowly (SGPs) and those that do not grow (NGPs) in the drug’s presence. At T36, after washing out voriconazole and replacing it with minimal medium, we assessed the recovery and growth of surviving conidia over an additional 36 h (Fig. 2a). This two-step approach allowed us to differentiate between immediate slow growth under drug pressure (T0) and the ability to resume growth after drug removal (T36) (Fig. 2a). T0 will reflect the strains that can grow slowly in the presence of VOR, named here as slow-growth persisters (SGPs), or do not grow at all, called here non-growth persisters (NGPs). The reference clinical isolate *A. fumigatus* A1160 is classified as an NGP (shown with a red arrow, Fig. 2a), and all the strains with T0 metabolic activity values that were ≤ than 2,000 were considered NGPs (see Table S1 at https://doi.org/10.6084/m9.figshare.31239700). In the acute patient isolates, there are 49 SGPs and 140 NGPs, while there are 81 SGPs and 131 NGPs in the chronic patient isolates. Post-hoc analysis comparing average metabolic activity at T0 and T36 revealed that chronic isolates exhibited significantly higher metabolic activity than environmental or acute isolates (*P* < 0.05; Fig. 2b), indicating an enrichment of small-colony phenotypes (SGPs) in this group. Using a metabolic activity threshold of >2,000 at T0 to define SGPs, chronic isolates contained 39% SGPs, compared to 23% in environmental and 25% in acute isolates. A χ^2^ test confirmed a significant association between isolate origin and SGP prevalence (χ² = 12.28, df = 1, *P* < 0.01). In the environmental isolates, there are 21 SGPs and 73 NGPs (Fig. 2a). There is a slight positive correlation (*P* = 0.075) between SGPs and chronic clinical isolates (Fig. 2e).

We established a persistence index (PI) by plating the conidia that were previously exposed to 96 h 4× MIC VOR on MM, counting the colony-forming units (CFUs), and dividing the CFUs by the original number of viable conidia (1.0 × 10^6^; Fig. 1b). As expected, the average PI for the top ten T0 SGPs is higher and significantly different from the average PI for the top 10 T0 NGP strains (Fig. 2c). However, the distribution of SGP CFUs differs from the distribution of NGP CFUs (Fig. 2d), and there is a non-significant trend (*P* = 0.075) (Fig. 2e).

### Genomic phylogenetics and variant evolution of the serial infection isolates

**Fig 3:**
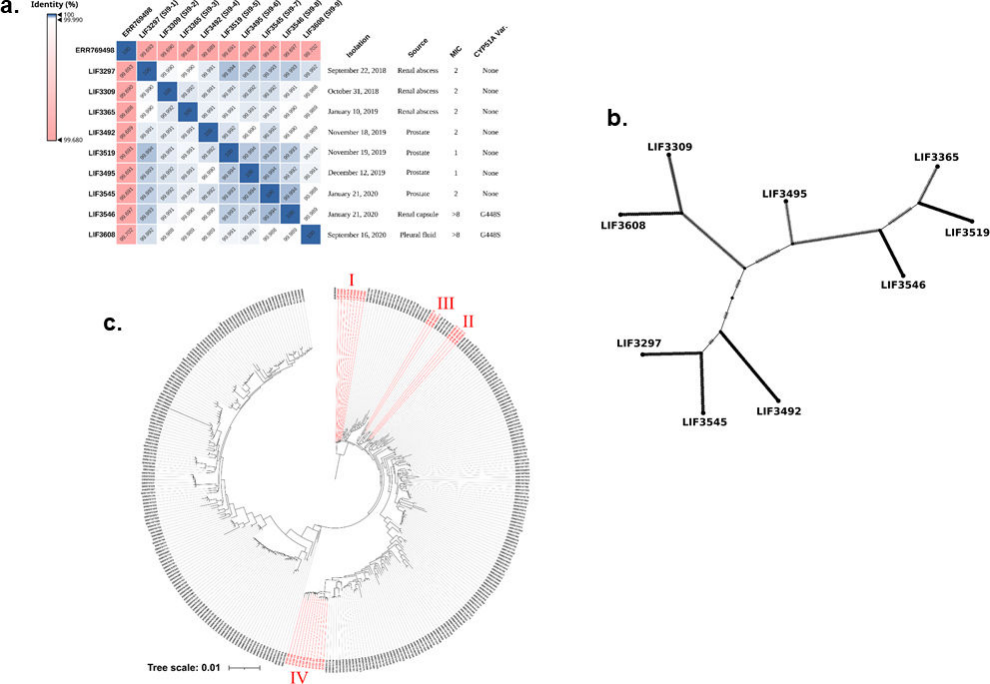
Evolutionary relationships among the serial isolates. (**a**) Average nucleotide identity between the isolate genomes, in addition to the closest external isolate according to the phylogeny, and the respective isolates’ origin information. (**b**) Phylogenetic tree with 318 *A. fumigatus*. I–IV in red, the four cases of serial isolates: I, SI9-1 to SI9-9 serial isolates; II and III, series from (22); and IV, series from (21). (**c**) Median-joining network based on haplotypes. The median-joining calculation and network generation were performed in PoPArt v.1.7 with epsilon = 0.

After the definition of two clear types of persistence in clinical and environmental isolates, we raised the hypothesis that *A. fumigatus* azole persistence could have evolved to increase the chances of survival until the fungus can evolve azole resistance, that is, increased azole persistence could precede azole resistance acquisition. Since we do not have the evolutionary history of each of the 495 isolates, we decided to look at a group of sequential isolates among these 495 that have evolved azole resistance. To learn more about the influence of persistence on VOR resistance, we identified nine isolates that were isolated from the same patient with aspergillosis over 2 years (Fig. 3a). In a timeline, seven of these first isolates were azole-susceptible and NGPs (Fig. 3a), while the other two latest isolates were azole-resistant (the serial isolates are here named serial isolates SI9-1 to SI9-9; Fig. 3a). A phylogenomic analysis was performed using 318 global *A. fumigatus* sequences, along with SI9-1 to SI9-9 and three other sets of serial isolates (21, 22), including newly sequenced and from previous studies (see Table S2 at https://doi.org/10.6084/m9.figshare.31239700), to assess the relatedness among the isolates in each series of four serial isolate sets from different studies (Fig. 3b). The phylogeny revealed that each of the four distinct groups of serial isolates (highlighted in red) forms a cluster in a single common ancestor among the isolates, suggesting that each series originated from a single infection event. Furthermore, average nucleotide identity (ANI) analysis revealed high genomic similarity among the nine serial isolates studied here (Fig. 3b), with significantly higher identity percentages when compared to the reference strain Af293 (assembled from the public data ERR769498), which coincidentally is the phylogenetically closest external isolate to this series (Fig. 3b), suggesting strong sequence similarity within the series.

The evolutionary relationships were evaluated among the nine serial isolates by constructing a median-joining haplotype network (Fig. 3c). The analysis revealed that genetic relatedness did not correlate with temporal isolation order, suggesting that *A. fumigatus* can undergo diversification within a host, forming distinct subpopulations over time. Haplotypes and phylogenomic analyses of these nine isolates suggested they belong to a common population (Fig. 3b and c). This population is composed of (listed in chronological order of isolation, from earliest to latest) LIF3297, LIF3309, LIF3365, LIF3492, LIF3519, LIF3495, LIF3545, LIF3546, and LIF3608 (these last two have evolved VOR resistance) (SI9-1 to SI9-9; Fig. 3a). Both VOR-resistant clinical isolates show the G448S mutation in their *cyp51A* coding regions, previously observed in previous studies (23, 24) (Fig. 3a). Variant calling of the nine serial isolates, using the strain Af293 as a reference, found 1,151 missense variants that changed during infection (see Tables S3 and S4 at https://doi.org/10.6084/m9.figshare.31239700), meaning that variants present in all sampled isolates were not considered in the analysis. Additionally, the analysis included disruptive variants (i.e., including indels, nonsense, or start-loss) for 267 genes (see Tables S3 and S4 at https://doi.org/10.6084/m9.figshare.31239700). These variants were included in a Venn diagram (see Fig. S1 at https://doi.org/10.6084/m9.figshare.31239700), created to identify variants potentially acquired during infection, comparing susceptible and resistant isolates. Based on SNP phylogeny (Fig. 3b) and the fact that the patient started using VOR (200 mg/12 h for 30 days) 26 days after the first hospitalization in 2018 (Fig. 3a), we excluded the first isolate in the temporal series (SI9-1, LIF 3297) to focus specifically on variants emerging after azole treatment began. Analysis of disruptive variants (see Fig. S1a at https://doi.org/10.6084/m9.figshare.31239700) revealed functional losses in five genes (resistant isolates) and 22 genes (susceptible isolates), with 3/5 and 18/22, respectively, showing parallel disruptive variants in other serial isolate groups (see Fig. S1b at https://doi.org/10.6084/m9.figshare.31239700). For missense variants, we focused our analyses on genes with at least three variants. This means that we prioritized genes in which we identified at least three distinct missense variants across the data set. It does not necessarily imply the variants are present in three different isolates but rather that the gene harbors multiple different missense mutations. The rationale behind this criterion is to focus on genes showing a higher degree of mutational burden, which may indicate selective pressure or functional importance related to the phenotype under study. By analyzing genes with multiple variants, we aim to reduce noise from random or isolated mutations and highlight candidates potentially involved in adaptation or persistence.

We found that the distribution of missense variants was concentrated on a few genes; these included 13 variants found exclusively in resistant isolates distributed in only 3 genes, 54 variants (susceptible isolates) distributed in 15 genes, and 41 variants (susceptible and resistant isolates) distributed in six genes. From these genes, only two did not show any variant that was also found in other groups of serial isolates (see Fig. S1b at https://doi.org/10.6084/m9.figshare.31239700). Some of these SNPs exclusive of the SI9-8 and SI9-9 isolates are present in genes that encode hypothetical or putative proteins, except for AFUA_5G06420, which encodes the Mitogen-Activated Protein Kinase Kinase Kinase (MPKKK) SteC (25) (see Fig. S1a at https://doi.org/10.6084/m9.figshare.31239700).

Taken together, these results indicate there is a single population in the nine serial isolates and the two VOR-resistant isolates in this population have evolved several unique SNPs. It remains to be validated if these SNPs contribute to the evolution of VOR resistance. The monophyletic relationship and the sequential accumulation of novel mutations across the isolates provide strong evidence for a single initial infection, followed by within-host diversification, effectively excluding a scenario of polyclonal infection from repeated environmental introductions.

### Serial isolates from the same patient have decreased susceptibility to stressful conditions and increased VOR persistence

These serial strains show diverse and heterogeneous growth phenotypes (see Fig. S2 at https://doi.org/10.6084/m9.figshare.31239700). The SI9-1 to -9 serial isolates also have progressively evolved decreased susceptibility to cell wall-damaging and oxidative-stressing agents (Table 1). SI9-1 to -9 serial isolates have variable but decreased susceptibility to bone marrow-derived macrophages (BMDMs) killing (see Fig. S3a at https://doi.org/10.6084/m9.figshare.31239700). However, susceptibility to A549 pulmonary epithelial cells is similar to that of the other serial isolates (see Fig. S3b at https://doi.org/10.6084/m9.figshare.31239700).

**TABLE 1:** MICs for the *A. fumigatus* serial isolates

Strain	CFW^*a*^ 25 µg/mL	CR^*a*^ 50 µg/mL	Menadione 0.01 mM	*t-*butyl 1 mM	H_2_O_2_ 5 mM
SI9-1 (LIF3297)	6.5	6.5	0.0025	0.015	5
SI9-2 (LIF3309)	6.5	6.5	0.0025	0.015	5
SI9-3 (LIF3365)	6.5	6.5	0.0025	0.015	5
SI9-4 (LIF3492)	12.5	12.5	0.005	0.03	5
SI9-5 (LIF3519)	6.5	6.5	0.0025	0.015	5
SI9-6 (LIF3495)	6.5	6.5	0.0025	0.015	5
SI9-7 (LIF3545)	6.5	6.5	0.0025	0.625	5
SI9-8 (LIF3546)	12.5	12.5	0.005	0.625	5
SI9-9 (LIF3608)	25	12.5	0.01	0.5	≥5

^*a*^ CFW, calcofluor white; CR, congo red.

**Fig 4:**
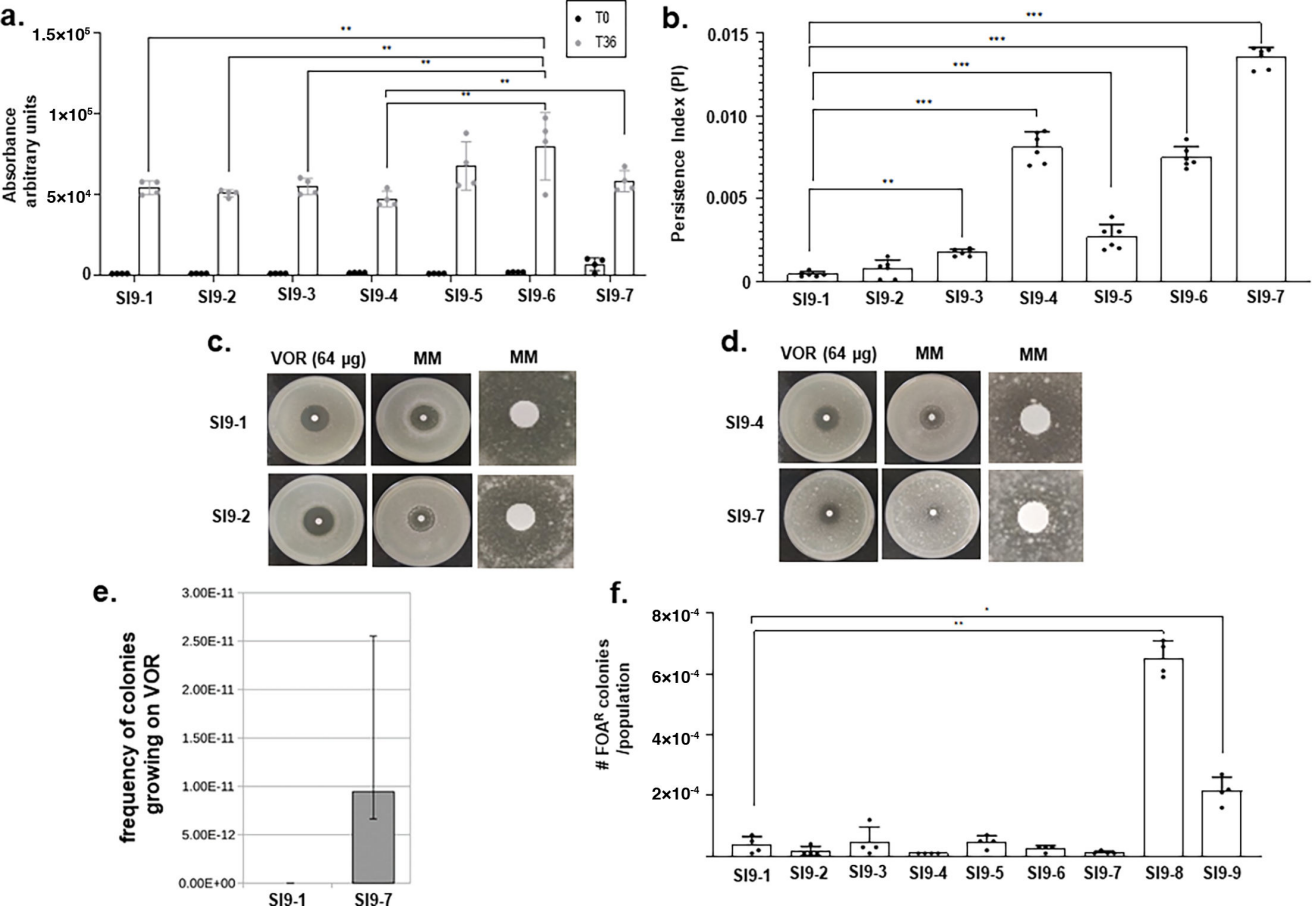
Characterization of the *A. fumigatus* serial isolates evolved in a single patient. (**a**) Metabolic activity of *A. fumigatus* serial isolates after incubation with supra-MIC concentrations (4× MIC) of VOR. The results represent the average of four repetitions ± standard deviation. (**b**) Persistence index of *A. fumigatus* serial isolates after incubation with supra-MIC concentrations (4× MIC) of VOR. The results represent the average of six repetitions ± standard deviation. (**c and d**) Growth in the inhibition halo of small colonies with increased VOR persistence. A sterile filter paper disk embedded with 10 µL of VOR (64 µg) was placed on a culture previously top-agar inoculated with *A. fumigatus* serial isolates. After incubation for 24 h at 37°C, the filter paper disk was replaced with another sterile filter paper disk embedded with 10 µL of MM and allowed to grow for an additional 24 h at 37°C. (**e**) Luria-Delbrück fluctuation test for *A. fumigatus* serial isolates SI9-1 and SI9-7. (**f**) Mutation frequency based on the number of 5-FOA-resistant mutants that can grow (plates containing 0.75 µg/mlL of 5-FOA). The results represent the median of 12 independent populations. *P < 0.05; **P < 0.01; and ***P < 0.001.

SI9-1 to -9 serial isolates are NGP (Fig. 1a), although SI9-7 (LIF3545) shows increased metabolic activity (Fig. 4a). However, SI9-1 to SI9-7 have increased survival in the presence of VOR (Fig. 4b), suggesting that increased persistence could be associated with the evolution of drug resistance. Disc diffusion assays also showed increased persistence in the SI9-2 and SI9-7 isolates when compared to the SI9-1 and SI9-4 isolates, respectively (Fig. 4c and d). For these experiments, we spread 10^4^ conidia of the isolates SI9-1, SI9-2, SI9-4, and SI9-7 on MM agar plates, placed a 6-mm disc in the center containing a final amount of 64 µg VOR, and incubated the plates for five days at 37°C. After this period, we replaced the 6-mm disc with a new one embedded with 10 µL of liquid MM and allowed them to grow for a further three days. In the first 5 days, the inhibiting halos showed consistent results. However, in the plates where the discs were replaced, the isolates SI9-2 and SI9-7 were able to form small colonies within the halo of inhibition, suggesting that a few conidia can germinate and have slow growth in the presence of supra-MIC VOR concentrations (Fig. 4c and d). In both PI and halo assays, we isolated 10 colonies per treatment that had emerged on MM + VOR or grown in the halo area and re-plated them on drug-free medium and on MM + VOR again to assess the stability of the phenotype. All the colonies failed to regrow on MM + VOR, supporting the conclusion that these were non-resistant persister cells.

*A. fumigatus* serial isolates SI9-1 and SI9-7 were assayed for frequency of colonies capable of growing on VOR by using a modified Luria-Delbrück fluctuation test (26, 27) for 8 µg/mL VOR (Fig. 4e). Twelve independent replicate cultures grown without selection were challenged on MM containing VOR to determine the probability that cells would spontaneously gain mutations that provide VOR resistance. On VOR, we did not observe any colony growth for SI9-1; in contrast, the clinical isolate SI9-7 had a spontaneous frequency of colonies capable of growing on VOR of 1.1 × 10^−11^ (Fig. 4e). In addition to the Luria-Delbruck fluctuation test, we used a 5-fluoroorotic acid (5-FOA)-based method, which is widely used to assess basal spontaneous mutation in fungal species (28, 29). By counting the number of 5-FOA-resistant mutants that can grow on the plates containing 0.75 µg/mL of 5-FOA, we found that the mutation frequency for SI9-8 compared to SI9-1 was about 20-fold higher, while SI9-9 to SI9-4 was about 10-fold higher (Fig. 4f). It is difficult based on the current data to estimate how the general mutation rate correlates with the VOR-specific mutation rate. However, it is clear from the FOA experiments that the SI9-8 and SI9-9 strains have increased basal spontaneous mutation rate.

Taken together, these results suggest that in their route to acquire VOR resistance, the serial isolates evolve several genetic traits that allow them to survive in more stressful conditions and persist in the presence of VOR.

### Transcriptional profiling of selected serial isolates

**Fig 5:**
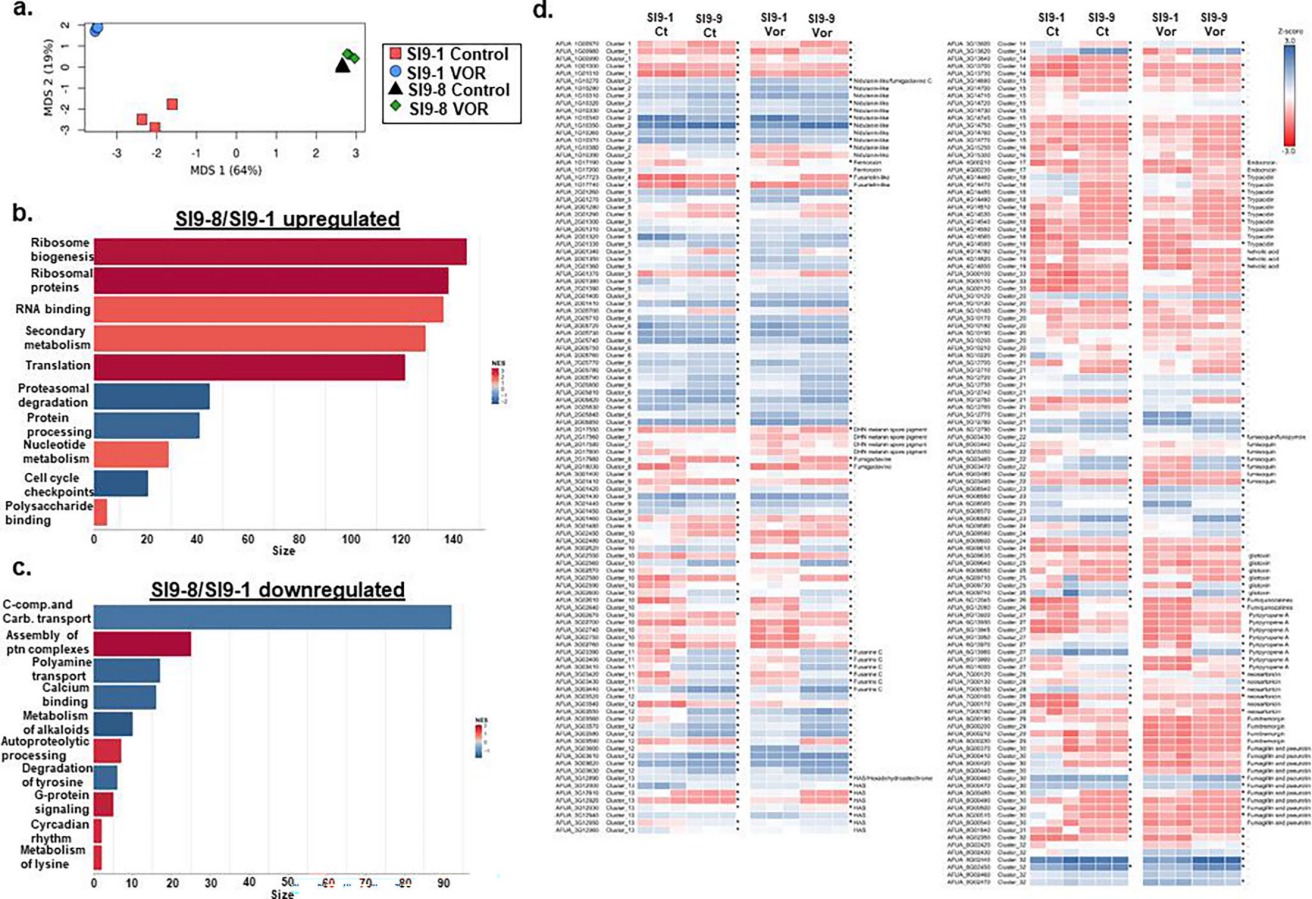
The SI9-8 isolate has a transcriptional signature distinct from the SI9-1 isolate. (**a**) PCA distribution of RNA-seq. (**b and c**) FunCat (Functional Categorization) using Gene Set Enrichment Analysis (GSEA; https://fungifun3.hki-jena.de/) for all these differentially expressed genes. (**d**) Heat map showing the expression of genes encoding proteins present in the BGC of SMs.

As an initial step to identify genetic traits that have evolved in these serial isolates that could affect transcriptional programs involved in increased VOR persistence and resistance, we evaluated the SI9-1 and SI9-8 isolates’ growth in liquid MM for 24 h without or with 0.5XMIC VOR. The transcriptional profiling was performed at 0.5× MIC voriconazole to capture early and subtle gene expression changes that occur under sub-inhibitory drug stress, which are often more informative about regulatory responses and adaptation mechanisms. In contrast, the 4× MIC concentration used in the other assays was intended to evaluate phenotypic tolerance and persistence under strong fungicidal pressure. There is no significant difference between SI9-1 and SI9-8 isolates grown 24 h in MM (SI9-1 = 0.120 ± 0.002 mg and SI9-8 = 0.132 ± 0.007 mg dry weight), but SI9-8 has about 4-fold more biomass than SI9-1 in MM+0.5 XMIC (SI9-1 = 0.035 ± 0.014 mg and SI9-8 = 0.146 ± 0.001 mg dry weight). In both experimental designs, we concentrate our attention on differentially expressed genes (DEGs) that have log2 scores of ≥ 1 and ≤ −1 and a statistically significant FDR value. PCA analysis showed that there is a clear difference between the DEGs of SI9-1 VOR and control, but DEGs of SI9-8 showed a similar distribution (Fig. 5a). We compared the differential expression of SI9-8 versus SI9-1 (Fig. 5b and c; Table S5, https://doi.org/10.6084/m9.figshare.31239700). In the absence of VOR, the SI9-8 versus SI9-1 showed 2,653 and 2,053 DEG down- and up-regulated, respectively (see Table S5 at https://doi.org/10.6084/m9.figshare.31239700). FunCat (Functional Categorization) using Gene Set Enrichment Analysis (30) (GSEA; https://fungifun3.hki-jena.de/) for all these DEGs showed a transcriptional up-regulation of genes coding for proteins involved in translation, secondary metabolism, proteasomal degradation, and cell cycle checkpoints (*P*-value < 0.01; Fig. 5b). There is a transcriptional downregulation for genes encoding proteins participating in C-compound and carbohydrate transport, assembly of protein complexes, polyamine transport, and calcium binding (Fig. 5c). There is a progressive increase in up-regulation of several genes present in different biosynthetic gene clusters (BGCs, genes in the BGCs DHN melanin spore pigment, hexadehydroastechrome [HAS], helvolic acid, fumisoquin, pyripyropene A, fumitremorgin, and fumagillin and pseurotin) that were transcriptionally modulated mainly in the SI9-8 strain (Fig. 5d). Most of these highly modulated BGCs are located in sub-telomeric regions (31) (see Fig. S4 at https://doi.org/10.6084/m9.figshare.31239700), raising the exciting hypothesis that SI9-8 has increased epigenetic regulation, allowing the derepression of these genes that are usually silenced due to the heterochromatic localization. Interestingly, there is an increased modulation in the SI9-8 strain of genes encoding proteins involved in chromosome metabolism, chromatin remodeling, and histone modification (see Fig. S5 at https://doi.org/10.6084/m9.figshare.31239700).

**Fig 6:**
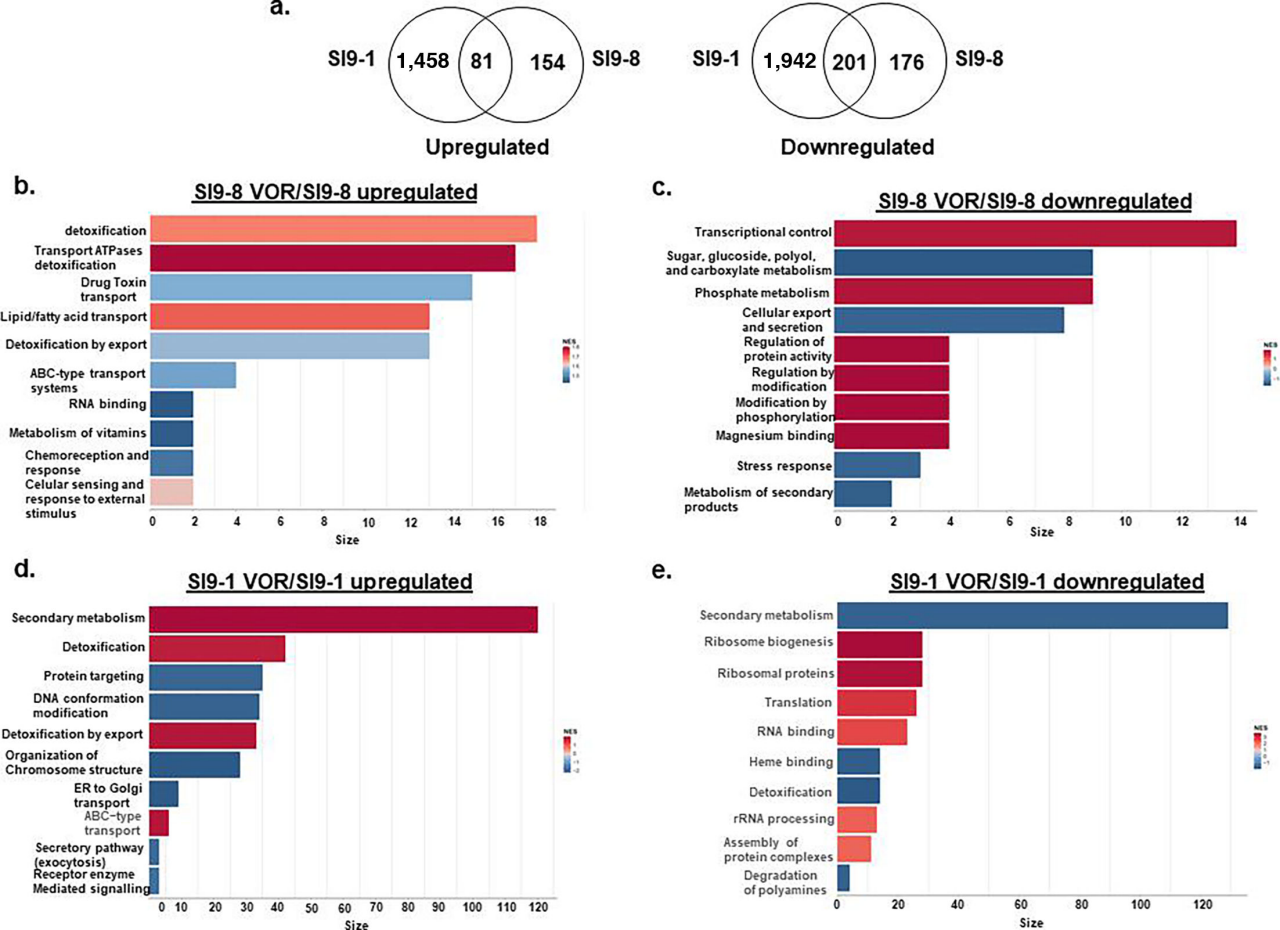
Genes differentially expressed in the SI9-1 and SI9-9 serial isolates, exposed or not to VOR. (**a**) Venn diagram. (**b–e**) FunCat (Functional Categorization) using Gene Set Enrichment Analysis (GSEA; https://fungifun3.hki-jena.de/) for all these differentially expressed genes.

We also looked at the transcriptional modulation of these serial isolates, but now grown in liquid MM for 24 h in the presence of 0.5XMIC VOR. We compared the differential expression of SI9-8 grown in the presence of VOR versus SI9-8 grown in the absence of VOR, and also SI9-1 exposed to VOR versus SI9-1. The SI9-8 VOR showed 235 and 377 DEG down- and up-regulated, respectively, while SI9-1 VOR showed 1,539 and 2,143 DEG down- and up-regulated, respectively (see Tables S6 and S7 at https://doi.org/10.6084/m9.figshare.31239700). Venn analysis showed that clinical isolates SI9-1 and SI9-8 strains share 81 upregulated genes, while SI9-1 and SI9-8 have 1,458 and 154 unique upregulated genes, respectively (Fig. 6a). SI-91 and SI9-8 share 201 downregulated genes, while SI9-1 and SI9-8 have 1,942 and 176 unique downregulated genes, respectively (Fig. 6a). FunCat (Functional Categorization) using Gene Set Enrichment Analysis (30) (GSEA; https://fungifun3.hki-jena.de/) analysis for all these DEGs showed that for SI9-1 VOR versus SI9-1, a transcriptional upregulation of genes coding for proteins involved in secondary metabolism, detoxification, DNA conformation modification, and organization of chromosome structure and a transcriptional downregulation of secondary metabolism, ribosome biogenesis, and translation (*P-*values < 0.01; Fig. 6b through e).

Among the unique genes differentially expressed in SI9-8 exposed to VOR, we observed several genes encoding protein kinases and phosphatases, transcription factors, and transporters that could be involved either in detoxification or reduced uptake of VOR (see Table S8 at https://doi.org/10.6084/m9.figshare.31239700). We also observed six genes of the ergosterol pathway (*erg24A*, *erg3*, *cyp51A*, *erg4A*, *erg24B*, and *erg25B*) as upregulated in the SI9-8 (see Table S8 at https://doi.org/10.6084/m9.figshare.31239700).

Collectively, our results suggest that the isolate SI9-8 has a transcriptional signature markedly different from SI9-1. SI9-8 has increased expression of genes encoding proteins involved in the translational machinery and the biosynthesis of SMs.

### Production of SM in the serial isolates

**Fig 7:**
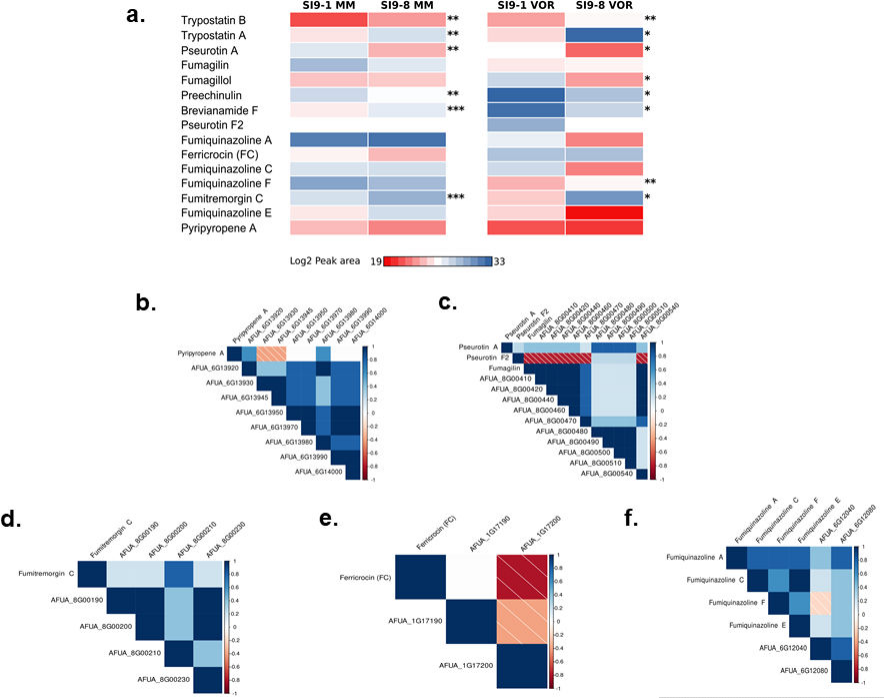
There is a partial correlation between the transcriptional profiling of BGC genes and the SM production. (**a**) Heatmap of metabolite abundance profiles in *A. fumigatus*. Average peak areas of metabolites are displayed as log2-transformed values across SI9-1 vs. SI9-8 in the conditions of minimum media and voriconazole. Significance levels (Student’s *t*-test, *P* < 0.05) are denoted by asterisks: * < 0.05; ** < 0.01; and *** < 0.001. (**b–f**). Correlation analysis between differentially expressed genes (DEGs) in the biosynthetic gene cluster (BGC) of *A. fumigatus* and related metabolomic profiles. Correlation matrix generated using corrplot in R, displaying Spearman’s rank correlation coefficients (ρ) between the FPKM values of BGC-associated DEGs and the peak areas of putative BGC-derived metabolites. Only significant correlations (*P* < 0.05) are shown. Positive correlations (blue) and negative correlations (red) are indicated with color intensity scaled by the strength of association (see gradient bar). Non-significant correlations (*P* ≥ 0.05) are painted in white.

We investigated the production of SM in the serial isolates S9-1 and S9-8 by using LC-MS/MS. Both strains were grown in the absence or presence of 0.5× MIC VOR for 5 days, and SMs were extracted. We were able to identify 15 SMs in both strains (Fig. 7a; Table S9, https://doi.org/10.6084/m9.figshare.31239700). In MM, SI9-8 showed a significant upregulation of trypostatins A and B, brevianamide F, and fumitremorgin C, while pseurotin A and preechinulin were downregulated compared to SI9-1 (Fig. 7a; Table S9, https://doi.org/10.6084/m9.figshare.31239700). This pattern suggests constitutive activation of the fumitremorgin and brevianamide biosynthetic pathways in later isolates, potentially contributing to enhanced fungal fitness during chronic infection. Notably, VOR exposure induced markedly different metabolic responses. While trypostatins A and B remained elevated and fumitremorgin C showed further upregulation, we observed significant induction of fumiquinazoline F specifically under VOR conditions (Fig. 7a; Table S9, https://doi.org/10.6084/m9.figshare.31239700). Conversely, pseurotin A, preechinulin, brevianamide F, and fumagillol were strongly suppressed in SI9-8 when challenged with voriconazole (Fig. 7a; Table S9, https://doi.org/10.6084/m9.figshare.31239700).

Integration of transcriptomic and metabolomic data reveals cluster-specific regulation patterns. Comprehensive analysis of BGC expression and corresponding metabolite production revealed distinct regulatory relationships across five major SM clusters in the serial *A. fumigatus* isolates (Fig. 7b through f). The pyripyropene A-associated BGC exhibited partial activation, with eight of nine genes showing differential expression. Among these, two genes demonstrated significant positive correlation with pyripyropene A production, while two others showed negative correlation, suggesting complex transcriptional regulation of this meroterpenoid pathway (Fig. 7b). Analysis of the 21-gene Fumagillin/Pseurotin supercluster revealed coordinated expression patterns, with 10 differentially expressed genes showing positive correlation with pseurotin A production (Fig. 7c). These same genes were positively correlated with fumagillin accumulation, while six genes exhibited negative correlation with pseurotin F2 levels, indicating divergent regulation of metabolic outputs within this supercluster (Fig. 7c). Four of nine genes in the fumitremorgin BGC were differentially expressed and positively correlated with fumitremorgin C production, demonstrating consistent transcriptional activation of this pathway (Fig. 7d). The ferricrocin cluster showed limited differential expression, with one of two DE genes exhibiting negative correlation (Fig. 7e). The fumiquinazoline cluster revealed metabolite-specific regulation patterns; while two DE genes showed positive correlation with fumiquinazolines A, C, and E, fumiquinazoline F production was associated with both positive and negative gene correlations (Fig. 7f).

Our results indicate that some SMs are produced independently of exposure to VOR in the SI9-8 clinical isolate and that many SMs are either induced or repressed by VOR in both clinical isolates. Moreover, there is a partial correlation between the transcriptional profile of BGC genes and SM production.

## DISCUSSION

Azoles inhibit lanosterol 14-α-demethylases encoded by the genes *cyp51A* and *cyp51B,* preventing the formation of ergosterol, a key component of fungal plasma membranes (32, 33). Initially, azole resistance was identified in *A. fumigatus* patients with chronic disease (34). Still, the most predominant azole-resistant strains are now derived from the agricultural environment, where massive amounts of triazole fungicides are applied (35). The most common resistance mechanism is related to variations in the target gene *cyp51A*, that is, point mutations in the coding sequence and a tandem repeat (TR) of 34 or 46 bases in the promoter region of *cyp51A* (TR34/Leu98His; TR46/Tyr121Phe/Thr289Ala), altering the protein structure and increasing the transcription and expression of the gene (33, 36). Treatment failure is common in patients infected with azole-susceptible isolates. This can result from several overlapping factors, such as poor drug absorption or azole persistence. Recently, we proposed a consensus definition of antifungal tolerance and persistence in filamentous fungi (14). Azole persistence describes the ability of *A. fumigatus* to survive drug action for extended periods upon exposure (9), which may have implications for the clinical treatment of infections.

Previously, azole persisters have been identified in two hematological patients with proven invasive aspergillosis (IPA) who experienced treatment failure despite infection with susceptible isolates, appropriate antifungal therapy, and therapeutic azole levels (6). Microbiological analysis revealed that both patients were infected with multiple strains, including persisters to VOR and/or isavuconazole. The authors propose that azole persistence may have contributed to therapeutic failure in these cases and suggest that this phenomenon warrants further investigation (6). The mechanism of action underlying *A. fumigatus* azole persistence is unknown. Preliminary evidence using a limited number of *A. fumigatus* environmental and clinical isolates suggested that a subpopulation of persister isolates can survive for extended periods and even grow at low rates in the presence of supra-MIC azole concentrations (19). Here, we describe a much broader study investigating the occurrence of VOR persistence in 495 clinical and hospital environmental isolates. Upon prolonged incubation in the presence of supra-MIC VOR concentrations, we observed two distinct behaviors: 30.5% of the population behaved like SGPs, while 69.5% behaved like NGPs. The SGPs are distributed across acute clinical and environmental isolates but are enriched in chronic clinical isolates. This can be explained by the fact that SGPs have more time to adapt and survive in patients with chronic aspergillosis who have prolonged azole exposure. While SGPs and NGPs differ in their *in vitro* growth dynamics on drug-containing medium, both phenotypes can survive VOR exposure and may represent alternative manifestations of persistence.

Among the 495 strains investigated, we identified a clinical case in which a single patient was treated for about 2 years with VOR, during which nine strains were isolated from this patient (24). Genome sequencing, phylogenomics, and haplotype analysis indicated that these nine strains belong to the same population, suggesting that this patient was infected in a single event. The seventh and eighth of these strains are VOR^S^ and VOR^R^, respectively, and were isolated on the same day from different clinical specimens, one from a prostate biopsy and the other from a renal capsule (see Fig. 3a for a timeline of the isolation). The eighth and ninth strains have acquired VOR^R^ by a G448S mutation in the *cyp51A* gene. The phylogenomic analysis, utilizing 318 global *A. fumigatus* sequences alongside four sets of serial isolates (including ours), demonstrates that each series forms a distinct monophyletic cluster. This clustering pattern strongly supports the hypothesis that each series originated from a single, unique infection event. Further reinforcing this observation, the average nucleotide identity (ANI) analysis revealed very high genomic similarity among the nine serial isolates studied here, indicating a high degree of relatedness. While the phylogenetic and clinical data are consistent with in-host diversification, we cannot completely exclude the possibility of a polyclonal infection involving closely related strains from a common environmental source.

The haplotype network analysis of our nine serial *A. fumigatus* isolates revealed that relatedness among the isolates did not correlate with their temporal isolation order, indicating that the pathogen undergoes significant diversification within the host over time, forming distinct subpopulations. The ability of *A. fumigatus* to generate distinct subpopulations, even within a relatively short time frame for clinical chronic infections rather than in absolute evolutionary terms, may contribute to its persistence and adaptability during chronic infections. These subpopulations could represent adaptations to varying micro-environments within the lung, differential immune pressures, or the emergence of drug-resistant variants. This observation aligns with the growing body of evidence demonstrating the capacity of *A. fumigatus* for micro-evolution during infection (21, 22, 37).

The very high genomic similarity of these strains and the fact that the last two have acquired a typical clinical azole-resistance mutation in the *cyp51A* coding region argue against the possibility of the patient being reinfected with azole-resistant strains from the agricultural environment, which usually harbor tandem repeat mutations in the *cyp51A* promoter regions. These results suggest that seven of these strains have survived the toxic azole patient environment for at least 18 months. Based on this, we hypothesized that the survival of these strains in the patient could be due to increased VOR persistence during this period. The seven VOR^S^ strains are NGPs and exhibit increased VOR persistence before developing VOR^R^. They have also developed decreased susceptibility to several stressing agents, such as cell wall damage and oxidative stressors. They have also increased survival rates in the presence of murine macrophages, but not in the presence of epithelial cells. The increased resistance to these agents suggests that persistence in these strains is not solely related to VOR but also to several other stressful conditions encountered by these strains in the patient. Interestingly, the last ones in the timeline of these strains have evolved an increased frequency of colonies capable of growing on VOR or FOA, as demonstrated by increased modified Luria-Delbrück fluctuation assays or basal spontaneous mutation rate. However, we cannot provide an explanation for the elevated basal mutation rate for VOR in isolate SI1-9, but not for 5-FOA. We have not identified any significant SNPs in genes involved in mismatch repair or other DNA repair systems that could explain this increased frequency. However, these strains exhibit increased resistance to oxidative stress agents, which could explain the higher frequency rates. This has previously been suggested for the temperature-induced mutagenesis of the pan-resistant *Rhodosporidiobolus* spp. (38), but whether that is the case for the *A. fumigatus* VOR^R^ evolution in the serial isolates remains to be demonstrated. However, the relationship between oxidative stress resistance and mutation rate is multifaceted and may depend on the interplay of survival advantage, DNA repair efficiency, and cellular metabolism, rather than a straightforward cause-and-effect. Further experimental work is needed to dissect these dynamics in detail.

Transcriptional profiling experiments revealed that the strain SI9-8 (a VOR^R^ serial isolate) shows increased mRNA levels for genes encoding proteins important for SM production. At least 33 BGCs have been identified in *A. fumigatus* (39). In our transcriptional profile, we observed complete or partial modulation of genes from all these BGCs in the SI9-8 clinical isolate. However, we identified only 15 SMs by LC-MS/MS, and 10 of them have the corresponding BGCs that are currently identified. These 10 BGCs showed a partial correlation between the transcriptional profile of their genes and SM production. These genes are part of BGCs located mainly in heterochromatic subtelomeric regions. Based on their location and the fact that SI9-8 is one of the last serial isolates in the timeline, we raised the hypothesis that heterochromatic regions are transcriptionally active in this strain, suggesting that increased VOR persistence not only precedes VOR^R^ but also affects epigenetic regulation. Although there is increased modulation of genes encoding chromatin and histone-modifying enzymes in the SI9-8 isolate, this hypothesis remains to be addressed. Increased aflatoxin production by *A. flavus* is linked to exposure to sublethal doses of azole fungicides (40, 41). Similarly, several studies have shown that *Fusarium graminearum* isolates adapted to tebuconazole produce higher amounts of toxins (trichothecenes) compared to non-adapted control strains (42, 43). In a clinical case study, aflatoxins B1, B2, and M1 were detected in lung lesion extracts from a patient with disseminated aspergillosis caused by *A. flavus*, who was treated with azole drugs (44). Further research is needed to understand how azole treatment may affect SM production and epigenetic regulation in filamentous fungi.

In addition to mutations in the *cyp51A* gene, an increasing number of azole-resistant isolates display resistance mechanisms caused by non-*cyp51A* mutations (45, 46). These mechanisms involve changes in various metabolic and transport pathways, such as upregulation of efflux pumps, modifications in enzymes essential for ergosterol biosynthesis, and alterations in mitochondrial function (47–51). The diversity of non-*cyp51A* resistance mechanisms complicates the overall resistance landscape and highlights the need for further investigation to fully understand their role in azole resistance. Interestingly, reports are showing the identification of non-*cyp51A* mutations that confer azole resistance in agricultural and clinical natural populations of *A. fumigatus* (52–54). We have also identified several unique non-CYP51A mutations in the VOR-resistant strains SI9-8 and SI9-9, and it remains to be determined whether these mutations contribute to the evolution of the azole resistance phenotype. It is tempting to speculate that these non-*cyp51A* azole-resistance mechanisms could be evolutionary alternatives in the trajectory to the acquisition of azole resistance. However, it is also possible to imagine that non-*cyp51A* azole-resistance mechanisms could be intermediary steps before the acquisition of *cyp51* mutations. This second hypothesis could explain why *cyp51A* mutations are predominantly observed in both agricultural and clinical settings. It remains to be determined whether, in natural populations undergoing azole-resistance acquisition, there is a concomitant increase in azole persistence, as we observed in the *A. fumigatus* serial isolates that evolved in a single patient.

A key question arising from our study is the mechanistic basis for the observed transition from persistence to resistance. While our data robustly demonstrate the temporal precedence of persistence, the causal links remain to be fully elucidated. Our multi-omics analyses provide a foundation for testable hypotheses, pointing to several pathways—including secondary metabolite (SM) production, chromatin remodeling, oxidative stress response, and general cellular homeostasis pathways like the cell wall integrity and HOG MAPK pathways—as potential modulators of the persistence phenotype. We hypothesize that genetic or epigenetic changes in these pathways not only enable survival under drug pressure but also create a permissive evolutionary landscape that facilitates the acquisition of canonical resistance mutations, such as the cyp51A G448S mutation. While our transcriptomic and metabolomic analyses revealed multiple pathways potentially linked to persistence, including oxidative stress and secondary metabolite biosynthesis, we did not identify a single validated genetic or biochemical mechanism. Future work will be needed to test the contribution of these pathways using gene deletion, overexpression, and chemical inhibition approaches. Our study thus defines a new paradigm for antifungal resistance and provides a roadmap for these critical future investigations.

## MATERIALS AND METHODS

### Media and strains

The *Aspergillus* spp. VOR-susceptible strains used in this work are listed in Table S1 at https://doi.org/10.6084/m9.figshare.31239700. Clinical isolates LIF3546 and LI3608 (here named SI9-8 and SI9-9), both VOR^R^, were obtained from a single patient as described by Pontes et al. (24). All *Aspergillu*s strains were grown in either solid MM (1% [wt/vol] glucose, 50 mL of a 20× salt solution, 1 mL of trace elements, 2% [wt/vol] agar, pH 6.5) or liquid MM (same composition as solid MM but without agar) at 37°C. The composition of the trace elements and nitrate salts is described by Käfer (55).

### Minimum inhibitory concentration and minimum effective concentration (MEC)

MIC and MEC values were determined according to the Clinical and Laboratory Standards Institute document M38-A3. Visual assessment was performed after a 48-h static incubation at 37°C. Antifungal agents (itraconazole, voriconazole, caspofungin, isavuconazole, posaconazole, and amphotericin B; Sigma-Aldrich) were tested across a concentration range of 0–4 µg/mL. Assays were conducted in 96-well flat-bottom polystyrene plates containing RPMI-1640 or MM, inoculated with 2.5 × 10⁴ conidia/mL. Following 48-h incubation at 37°C, plates were examined visually. For itraconazole, voriconazole, isavuconazole, posaconazole, and amphotericin B, the MIC endpoint was defined as the lowest concentration resulting in 100% growth inhibition relative to the drug-free control. The MEC for caspofungin was evaluated visually after 24 and 48 h of incubation. The endpoint was identified as the lowest concentration producing abnormal, compact, and rounded hyphal forms compared to the positive growth control. *A. fumigatus* strain A1160 was included as a quality control in all susceptibility tests.

### Alamar Blue assay

Antifungal activity of VOR was assessed against *A. fumigatus* strains using an Alamar Blue (Life Technologies) metabolic assay. Screening was performed in 96-well microplates. Each well contained 100 µL of liquid MM inoculated with 2.5 × 10⁴ conidia/mL, supplemented or not with VOR at an inhibitory concentration, along with a 10% volume of Alamar Blue reagent. Plates were incubated for 48 h at 37°C. Control wells consisted of medium alone (positive control) and medium with Alamar Blue but without conidia (negative control). Following incubation, fluorescence was measured (excitation 570 nm, emission 590 nm) using a SpectraMax Paradigm Multi-Mode Microplate Reader (Molecular Devices). Data, expressed as mean ± standard deviation (SD), were derived from multiple independent experiments conducted in duplicate. NGPs were classified as clinical isolates exhibiting a metabolic activity signal (T0) ≤ 2,000.

### Environmental sampling

Air samples were collected from indoor patient rooms and bathrooms at Hospital de Clínicas da UNICAMP (HC-UNICAMP) across summer, autumn, and spring. A Bio Samp Model MBS 1000D air sampler (Yotsubishi Corp., Japan) was used, drawing volumes of 1,000 L or 500 L at a height of approximately 1.5 m. Triplicate samples were taken in rooms, with single samples from bathrooms. Concurrent temperature and humidity readings were recorded. Sampled air was impinged onto Sabouraud Dextrose Agar (Difco, USA), with plates incubated at 37°C for 72 h. Fungal colonies displaying micro- and macromorphology consistent with *A. fumigatus* were purified. Isolates selected for the working stock were preserved in distilled water.

### RNA purification and preparation for RNA-seq

A total of 10^6^ spores/mL of *A. fumigatus* SI9-1 and SI9-8 clinical isolates was inoculated in 50 mL of liquid MM at 37°C for 24 h under agitation in the absence or presence of 0.5× MIC VOR. Total RNA was extracted by the TRIzol method, treated with RQ1 RNase-free DNase I (Promega), and purified using the RNAeasy kit (Qiagen) according to the manufacturer’s instructions. The total RNA was quantified using a NanoDrop, and its integrity was analyzed using an Agilent 2100 Bioanalyzer. All RNA had a minimum RNA integrity number (RIN) value of 8.0. For RNA sequencing, the cDNA libraries were constructed using the TruSeq Total RNA with Ribo Zero (Illumina, San Diego, CA, USA). From 0.1 to 1 µg of total RNA, the ribosomal RNA was depleted, and the remaining RNA was purified, fragmented, and prepared for complementary DNA (cDNA) synthesis, according to the manufacturer’s recommendations. The libraries were validated following the Library Quantitative PCR (qPCR) Quantification Guide (Illumina). Following the RNA-seq was carried out by paired-end sequencing on the Illumina NextSeq 500 Sequencing System using NextSeq High Output (2 × 150) kit, according to the manufacturer’s recommendations.

### Differential expression analysis

The quality of sequences was assessed using FastQC v0.11.8 (54). Adapter sequences were trimmed, and reads were quality-filtered using Trimmomatic v0.39 (56) with the parameters ‘LEADING:20 TRAILING:20 MINLEN:60.’ The filtered reads were aligned to the *A. fumigatus* reference sequence Af293 (NCBI assembly code GCA_000002655.1) using Hisat v2.2.1 (57) with splice site positions and a maximum intron length of 3,093, both obtained from the reference Af293. Subsequently, the SAM files were converted to BAM format and sorted using SAMtools v1.15.1 (58). FeatureCounts v1.5.3 (59) was used to quantify non-ambiguous aligned fragments and produce tables for RNAseq analysis. Differential expression analysis of the filtered genes was performed using edgeR v3.14.0 (60) with the simple design protocol (61), where the counts were converted to counts per million (CPM), and features were filtered, ensuring that the sum of all samples had a CPM of at least 3, considering samples with a CPM above 1 for the sum.

### Genomic data acquisition

To expand our data set, we incorporated publicly available whole-genome sequencing (WGS) data of *A. fumigatus* from previous studies, which are composed of data from strains serially isolated from patients (NCBI project accession numbers: PRJDB3064 and PRJNA528395) and 318 isolates from nine studies (NCBI project accession numbers: PRJEB27135, PRJDB10244, PRJEB8623, PRJNA638646, PRJNA798608, PRJNA592352, PRJNA548244, PRJNA961646, and PRJNA592352). Raw reads were retrieved from GenBank (62) using SRA Toolkit v3.0.2 (63). The quality of sequences was assessed by FastQC v.0.11.8 (54), followed by adapter trimming and quality filtering done with Trimmomatic v0.39 (56) using parameters “ILLUMINACLIP: NexteraPE-PE.fa:2:30:10 HEADCROP:9 LEADING:20 TRAILING:20 MINLEN:60.”

### Genome assembly and annotation

*De novo* genome assemblies were done using SPAdes v.3.14.0 (64). Genome completeness was inferred with BUSCO v5.4.4 (65) using *eurotiales_odb10* as the lineage parameter. The genomes were annotated using automatic protein prediction with AUGUSTUS v3.5.0 (66) with the parameter “–species=aspergillus_fumigatus.” The average nucleotide identity between the assembled genomes and the reference genome Af293 was calculated with FastANI v1.34 (67).

### Variant calling and SNP phylogeny

Processed reads were mapped to the reference genome of *A. fumigatus* Af293 (NCBI assembly code GCA_000002655.1) using Bowtie v2.3.5.1 (68). SAM files were converted to BAM format with SAMtools v1.15.1 (58). Single-nucleotide polymorphisms (SNPs) were identified and genotyped using the Genome Analysis Toolkit (GATK) v4.3.0.0 (69). The VCF files with haplotypes were created from BAM files using the module “HaplotypeCaller.” These files were merged into one using the “CombineGVCFs” module, with a combined collection of all the WGS data used in this study and the collection of nine serial isolates. These merged files were genotyped using the module “GenotypeGVCFs,” and then, low confidence SNPs were filtered out using the module “VariantFiltration” with the parameters “QualByDepth < 2.0 || MappingQuality < 40.0 || MappingQualityRankSumTest < −12.5 || FisherStrand > 8.0 || StrandOddsRatio > 3.0 || ReadPosRankSumTest < −2.0 || ReadPosRankSumTest > 2.0.” VCF files were annotated based on the reference strain Af293 using SnpEff (70). The final combined VCF file was converted to PHYLIP format using vcf2phylip v2.0 (Available in: https://zenodo.org/record/2540861) for phylogenetic analysis. High-confidence SNPs were used for the inference of the maximum likelihood tree, inferred with IQ-TREE v1.7 (71) using default parameters; the substitution model TVM+F + G4 was automatically selected, and the ascertainment bias correction (+ASC) was applied. The generated tree was edited in iTOL v6 (72). For analyses requiring per-sample variant calls, the sample gVCF files were also individually genotyped using GenotypeGVCFs and filtered using the same criteria described above to generate high-confidence VCF files for each isolate.

### Sample preparation and untargeted LC-HRMS analyses

SMs produced by different clinical isolates SI9-1 and SI9-8 in liquid MM with or without 0.5× MIC VOR were evaluated based on the dry weight of these isolates after 5 days of growth; 50 mg of dry weight from each isolate’s samples was resuspended in 1 mL of HPLC-grade methanol (MeOH), followed by ultrasonic bath extraction for 1 h. The samples were then centrifuged at 14,000 rpm for 15 min, and 700 µL of the supernatant was filtered through a 0.22 µm PTFE filter, transferred to vials, and diluted with HPLC-grade MeOH to a final volume of 1 mL.

LC-HRMS/MS analyses were performed in positive ionization mode using a Thermo Scientific QExactive hybrid Quadrupole-Orbitrap mass spectrometer coupled to a Dionex UltiMate 3000 RSLCnano HPLC system. A Bruker Intensity Solo 2 C18 column (100 × 2.2 mm, 1.8 µm) was used as the stationary phase. The column temperature was set at 40°C. Mobile phase A consisted of H_2_0 with 0.1% formic acid, and mobile phase B consisted of acetonitrile with 0.1% formic acid. The flow rate was set at 250 µL/min, with 10 µL of samples injected. The 24-min chromatographic run started at 5% solvent B and increased as follows: 5 min, 5%; 10 min, 40%; 12 min, 45%; 18 min, 98%; 20 min, 98%; 22 min, 5%; and 24 min, 5% solvent B. MS spectra were acquired over an *m/z* range of 100–1,500 Da, with a mass resolution of 75,000 at 200 Da. Ionization parameters included a sheath gas flow rate of 45 L/h, an auxiliary gas flow rate of 10 L/h, a sweep gas flow rate of 2 L/h, a spray voltage of 3.5 kV, a capillary temperature of 250°C, an S-lens RF level of 50, and an auxiliary gas heater temperature of 400°C. MS/MS spectra were acquired in DDA mode with normalized collision energies of 20, 35, and 45 V applied stepwise. The six most intense precursors per cycle were measured with a resolution of 17,000 at 200 Da.

### LC-HRMS/MS data processing

The LC-HRMS/MS data processing workflow for the clinical isolates was performed as described in (73). Raw chromatograms from the samples (.raw) were converted to the .mzXML format using the MSConvert tool from ProteoWizard (version 3.0.23,011) (74). The MZmine software (v.4.5.0) (75) was used for processing the converted .mzXML data with the following pre-processing parameters: Crop retention time (0.3–13.00 min), RT tolerance (0.10 min), MS1 noise level (2 × 10^6), MS2 noise level (1.0 × 10^3), minimum feature height (4.0 × 10^6), and *m/z* tolerance (5.0 ppm). The generated feature tables were exported in .csv format, and the MS/MS matches of the features detected in the samples were exported in .mgf format. Feature-based molecular networking (FBMN) was performed according to the workflow outlined in GNPS (https://ccms-ucsd.github.io/GNPSDocumentation/) (76). For spectral processing, .mgf files were filtered to retain only the five most intense fragmentation ions within a ±5 ppm mass window. Precursor and MS/MS fragment ion mass tolerances were set to 0.02 Da. Spectral matches between the network and reference libraries were filtered, requiring a score threshold >0.7.

Fungal secondary metabolites (SMs) and other annotated metabolites were categorized per the confidence levels defined by Schymanski et al. (77): Level I (structure confirmed by reference standard comparison), Level II (probable structure based on MS/MS spectral match to a library such as GNPS), Level III (tentative structure proposed from in silico-generated MS/MS evidence), and Level IV (unequivocal molecular formula assignment from LC-HRMS data).

Finally, the areas corresponding to the annotated SMs were calculated manually to determine their production in the different clinical isolates in MM and the presence of VOR, using the FreeStyle v.**1.8.63.0** SP2 software (Copyright © 2021 Thermo Fisher Scientific Inc.). In addition, the calculated peak areas were normalized by the dry weight of the samples used during sample preparation.

### A549 human lung epithelial cell cytotoxicity assay

*A. fumigatus* at a multiplicity of infection (MOI) of 1:10 (fungal cell:host cell). Following a 24-h co-incubation at 37°C with 5% CO₂, the culture medium was aspirated, and each well was washed three times with 1× PBS. To lyse the epithelial cells and recover fungi, 1 mL of sterile, cold water was added to each well. The resulting suspension was used for colony-forming unit (CFU) quantification. For CFU enumeration, 100 µL of the lysate (or a 1:100/1:1,000 dilution thereof) was plated onto YAG agar and incubated at 37°C for 4 days. A 50 µL aliquot of the original fungal inoculum (1 × 10³ cells/mL) was also plated to serve as a baseline for CFU count normalization. All conditions were assayed in triplicate. The percentage of surviving CFUs was calculated, and statistical analysis was performed using GraphPad Prism (GraphPad Software, Inc.). A *P*-value <0.0001 was considered statistically significant.

### Macrophage internalization and killing assay

Bone marrow-derived macrophages (BMDMs) were seeded at 10⁶ cells/well in 96-well plates. Cells were challenged with conidia from *A. fumigatus* clinical isolates at a multiplicity of infection (MOI) of 1:10 and incubated for 24 h at 37°C with 5% CO₂. Post-incubation, the medium was aspirated, and the wells were washed with ice-cold PBS. Macrophages were lysed using 50 µL of sterile water containing 0.1% (vol/vol) Triton X-100. The lysate was immediately diluted 1:200, and 50 µL was plated on Sabouraud agar. Plates were incubated overnight at 37°C for colony enumeration. A 50 µL aliquot of the initial inoculum (10³ spores/mL) was also plated to enable CFU count normalization. CFU/mL for each isolate was calculated and compared against the wild-type strain A1160.

### Modified Luria-Delbrück fluctuation assay

Fluctuation assays were performed based on established protocols with modifications (78, 79). Twelve independent *A. fumigatus* colonies were isolated from a minimal medium (MM) agar stock and grown for 4 days on MM plates at 37°C. Conidia were harvested in sterile water containing 0.01% Tween-20. A total of 1 × 10^10^ conidia were plated onto media containing voriconazole or COL + CAS, followed by incubation at 37°C for 10 days. Colonies were counted after 4 days (control RPMI-1640 medium) or 14 days (drug-containing media). Data analysis utilized the R package *flan* v0.9 (78) with default parameters, applying the maximum likelihood method to estimate mutation probability with a 95% CI.

### Clinical case

The clinical case was reported by Pontes et al. (24). In summary, the patient had undergone a heart transplant, was immunocompromised, and was undergoing recurrent immunosuppression therapies due to transplant rejection. He was hospitalized, reporting pain in the abdomen; after a CT scan, prostate and kidney involvement were seen. Initially, micafungin was used for 14 days until *A. fumigatus* was identified as the etiologic agent; subsequently, the patient was treated with VOR 200 mg/twice/day for 93 days. The patient was not responding to treatment, and itraconazole 200 mg/twice/day was introduced for 102 days, with the clinical condition worsening; hence, VOR 200 mg/twice/day was used again for ~150 days. After this period, liposomal amphotericin B was used for 33 days, and with the improvement in the condition, posaconazole + VOR 200 mg/twice/day was used for ~95 days. With the worsening of the clinical condition and pulmonary involvement, therapy with liposomal Amphotericin B was resumed for another 30 days, and the patient was discharged and died of COVID-19 after a few months.

### Voriconazole persistence index

Voriconazole persistence was quantified via colony-forming unit (CFU) recovery. For each strain, 200 µL of minimal medium (MM) containing 5 × 10⁶ CFU/mL conidia and voriconazole at 4× MIC was aliquoted into wells of a 96-well plate. Following a 96-h incubation at 37°C, wells were washed twice with sterile water, and the contents were resuspended in 100 µL of water. The entire suspension was then spread onto solid MM plates (drug-free). CFUs were enumerated after 48 h of incubation at 37°C. Each assay included a minimum of six technical replicates and was repeated in three independent experiments. Strain viability was confirmed by plating 100 CFU of fresh conidia onto solid MM and counting colonies after 48 h at 37°C. The assay was validated by determining the azole persistence of the parental strain A1160 and a known high-persister strain, PDE-9. The persistence index was calculated as: Persistence index = (CFUs recovered after azole exposure)/(initial conidial inoculum).

### Statistical analysis

Graph generation and statistical analyses were conducted using GraphPad Prism 10. Comparisons against wild-type or control conditions utilized a one-tailed paired *t*-test, one-way ANOVA, or two-way ANOVA, as appropriate.

## Data Availability

The WGS and RNA-sequencing data were submitted to NCBI under the BioProject ID PRJNA1254308. The final VCF files obtained from the variant call of serial isolates are available in FigShare (https://doi.org/10.6084/m9.figshare.29236379.v1). The pipeline and scripts used for the variant call analysis are available in GitHub (https://github.com/DelbajeEndrews/var_call). All the strains described here (described in Table S1, https://doi.org/10.6084/m9.figshare.31239700) are available upon request.
